# Seasonal Variation in Prevalence of *Mycoplasma hyopneumoniae* and Other Respiratory Pathogens in Peri-Weaned, Post-Weaned, and Fattening Pigs with Clinical Signs of Respiratory Diseases in Belgian and Dutch Pig Herds, Using a Tracheobronchial Swab Sampling Technique, and Their Associations with Local Weather Conditions

**DOI:** 10.3390/pathogens10091202

**Published:** 2021-09-16

**Authors:** Frédéric A. C. J. Vangroenweghe, Olivier Thas

**Affiliations:** 1 Business Unit Swine & Ruminants, Elanco Animal Health, Plantijn en Moretuslei 1A, 2018 Antwerpen, Belgium; 2 Unit of Porcine Health Management, Faculty of Veterinary Medicine, Ghent University, Salisburylaan 133, 9820 Merelbeke, Belgium; 3 I-BioStat, Data Science Institute, Campus Diepenbeek, Hasselt University, Agoralaan Gebouw D, 3590 Diepenbeek, Belgium; olivier.thas@uhasselt.be; 4 Department of Applied Mathematics, Computer Science and Statistics, Faculty of Sciences, Ghent University, Krijgslaan 281, 9000 Ghent, Belgium; 5 National Institute of Applied Statistics Research Australia (NIASRA), University of Wollongong, Northfield Ave, Wollongong, NSW 2522, Australia

**Keywords:** PRDC, tracheobronchial swabs, prevalence, swine

## Abstract

Besides *Mycoplasma hyopneumoniae* (*M. hyopneumoniae*), many other viruses and bacteria can concurrently be present in pigs. These pathogens can provoke clinical signs, known as porcine respiratory disease complex (PRDC). A sampling technique on live animals, namely tracheobronchial swab (TBS) sampling, was applied to detect different PRDC pathogens in pigs using PCR. The objective was to determine prevalence of different PRDC pathogens and their variations during different seasons, including correlations with local weather conditions. A total of 974 pig farms and 22,266 pigs were sampled using TBS over a 5-year period. TBS samples were analyzed using mPCR and results were categorized and analyzed according to the season of sampling and local weather data. In samples of peri-weaned and post-weaned piglets, influenza A virus in swine (IAV-S), porcine reproductive and respiratory syndrome virus—European strain (PRRSV1), and *M. hyopneumoniae* were found as predominant pathogens. In fattening pigs, *M. hyopneumoniae*, porcine circovirus type 2 (PCV-2) and PRRSV1 were predominant pathogens. Pathogen prevalence in post-weaned and finishing pigs was highest during winter, except for IAV-S and *A. pleuropneumoniae*, which were more prevalent during autumn. Associations between prevalence of several PRDC pathogens, i.e., *M. hyopneumoniae*, PCV-2 and PRRSV, and specific weather conditions could be demonstrated. In conclusion, the present study showed that many respiratory pathogens are present during the peri-weaning, post-weaning, and fattening periods, which may complicate the clinical picture of respiratory diseases. Interactions between PRDC pathogens and local weather conditions over the 5-year study period were demonstrated.

## 1. Introduction

Porcine respiratory disease complex (PRDC) is a multifactorial and complex disease in nursery and growing pigs [1], provoked by a combination of several infectious viral and bacterial pathogens, environmental stressors, differences in production systems, and management practices [2,3,4]. The disease, characterized by pneumonia and reduced growth performance, is an economically significant respiratory disorder of weaned piglets and finishing pigs, and remains a challenge to the swine industry worldwide. Multiple agents were reported to be associated with PRDC, including the major pathogens porcine reproductive and respiratory syndrome virus (PRRSV), *Mycoplasma hyopneumoniae* (*M. hyopneumoniae*), influenza A virus in swine (IAV-S), and porcine circovirus type 2 (PCV-2) [5,6]. Other pathogens associated with PRDC are porcine cytomegalovirus (PCMV), porcine respiratory coronavirus (PRCV), and *Actinobacillus pleuropneumoniae* (*A. pleuropneumoniae*) [2,5,6]. Infection with each single pathogen does not necessarily result in appearance of symptoms, but complex infections with a variety of pathogens can develop severe conditions.

*Mycoplasma hyopneumoniae* plays a major role within PRDC as the etiological agent of enzootic pneumonia, a chronic respiratory disease that mainly affects finishing pigs [7,8]. Mycoplasma infections are related to a chronic, non-productive cough. This results in economic losses due to reduced growth rate, poorer feed conversion, increased medication use and a higher susceptibility to secondary pathogens, such as *Pasteurella multocida* (*P. multocida*) and *A. pleuropneumoniae* [7]. Moreover, *M. hyopneumoniae* may act as a facilitator to other primary pathogens such as PRRSV [9,10], IAV-S [2,11,12], and PCV-2 [13,14]. The respiratory form of PRRSV primarily affects growing and finishing pigs, causing interstitial pneumonia, which induces respiratory signs [15]. PRRSV increases the susceptibility of pigs to secondary bacterial and viral infection [16,17,18,19,20,21]. Concurrent infections with PRRSV, PCV-2, and *M. hyopneumoniae* have been associated with more severe disease and higher mortality [17,22,23,24]. Swine influenza is mainly caused by influenza type A viruses and several subtypes of IAV-S have become enzootic in the pig population. Indeed, three IAV-S subtypes, namely H1N1, H2N1, and H3N2, currently circulate among pigs worldwide [25,26]. The enzootic within-farm persistence of IAV-S has recently been described as consecutive waves of diverse intensity in some Spanish farrow-to-finish operations [27]. Recently, pigs with passive immunity to IAV-S have been identified as potential disseminators of IAV-S, despite a potential reduction in clinical disease implied by this immunity [28]. Porcine circovirus type 2 is also responsible for considerable economic losses in the swine industry worldwide [29]. PCV-associated disease (PCVAD) can manifest as enteric, respiratory, reproductive, and systemic disease [30]. PCVAD is characterized by lymphoid depletion, which is considered the hallmark lesion [31]. This is thought to induce immunosuppression or immunomodulation in the host [32], leading to secondary infections with other viral or bacterial pathogens [33,34,35]. A field study in Spain confirmed detection of PCV-2 in several types of respiratory samples [36]. Porcine respiratory coronavirus (PRCV) is a naturally occurring respiratory variant of transmissible gastroenteritis virus in pigs, leading to fever and atypical pneumonia [37]. Seroprevalence of PRCV in young fattening pigs in Belgium varied from 34% during winter to 50% during late summer and autumn [38]. Dual infections involving PRCV and PRRSV or IAV-S did only identify little to no interactions between pathogens [39]. Porcine cytomegalovirus infection is endemic in the pig population [40]. Virus transmission occurs horizontally through nasal and ocular secretions, milk, and urine. *Actinobacillus pleuropneumoniae*, the etiological agent of pleuropneumonia in pigs, is the most important bacterial pulmonary pathogen in pigs worldwide. In its most virulent form, *A. pleuropneumoniae* induces severe, rapidly fatal pleuropneumonia in naïve pigs of all ages [41]. Virulence of *A. pleuropneumoniae* strains varies remarkably, ranging from acute disease with high mortality to more chronic respiratory problems without significant mortality. Animals may be carriers of *A. pleuropneumoniae* at the level of the tonsils and in chronic lung lesions.

Using conventional necropsy findings or serology is a relevant first step towards diagnosis of the complex combination of several PRDC pathogens. However, due to the absence of pathognomonic lesions involved and the variable interval between infection and seroconversion for each of these pathogens, it is frequently not easy to obtain a conclusive diagnosis. Therefore, direct detection of pathogens present through PCR techniques is currently used [5,6,42,43]. A validated sampling technique using tracheobronchial sampling [43,44,45,46] applied for early detection of *M. hyopneumoniae* in pigs [47] was used to collect samples in pigs of different age categories, with clinical signs of respiratory diseases, for subsequent analysis with a multiplex PCR (*M. hyopneumoniae*, PRRSV (PRRSV1, European strain; PRRSV2, North-American strain; PRRSV1,2, combined European and North-American strain), IAV-S, PCV-2, PRCV, PCMV), and a supplementary bacterial PCR (*A. pleuropneumoniae*), to detect seven different PRDC pathogens.

The aim of the present study was to detect the prevalence of several respiratory pathogens in pigs with clinical signs of respiratory disease using the TBS sampling technique and to gain insights into seasonal variation and correlation with local weather conditions.

## 2. Results

### 2.1. Prevalence of M. hyopneumoniae and Other PRDC Pathogens among Age Categories

**Figure 1:**
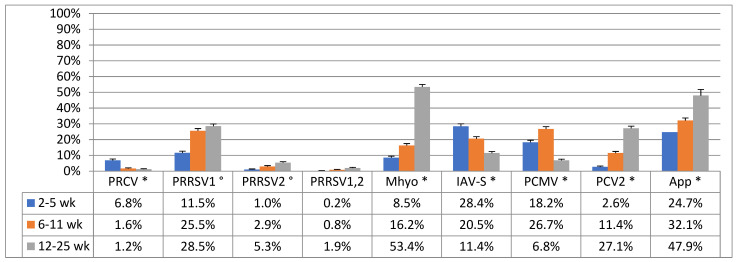
Prevalence (expressed as % positive samples ± SEM) of different single PRDC pathogens at different age categories, namely peri-weaned piglets (3–5 weeks of age), post-weaned piglets (6–11 weeks of age), and fattening pigs (12–25 weeks of age). In total, 22,266 pigs were sampled. PRCV, porcine respiratory coronavirus; PRRSV1, European strain of porcine reproductive and respiratory syndrome virus; PRRSV2, North-American strain of PRRSV; PRRSV1,2, combined European and North-American strain of PRRSV; *Mhyo*, *Mycoplasma hyopneumoniae*; IAV-S, influenza A virus in swine; PCMV, porcine cytomegalovirus; PCV-2, porcine circovirus type 2; App, *Actinobacillus pleuropneumoniae*. Significant differences (*p* < 0.05) in pathogen prevalence are indicated by superscript: * differences between all three age categories significant; ° differences between 2–5 weeks and 6–11 weeks of age, and between 2–5 weeks and 12–25 weeks.

The prevalence data of *M. hyopneumoniae* and all other PRDC pathogens among the different age categories are given in Figure 1. At 3–5 weeks of age, 8.5% of piglets were already *M. hyopneumoniae*-positive, increasing to 16.2% at 6–11 weeks of age. In the fattening period, sampled pigs with clinical signs of respiratory disease were 53.4% *M. hyopneumoniae*-positive.

During the peri-weaning period (3–5 weeks of age), most prevalent PRDC pathogens were IAV-S (28.4%), PCMV (18.2%), PRRSV1 (11.5%), and PRCV (6.8%). Other pathogens, such as PRRSV2, PRRSV1,2, and PCV-2 were much less prevalent. The secondary pathogen *A. pleuropneumoniae* occupied an intermediate position with a prevalence of 24.7% at 3–5 weeks of age.

During the post-weaning period (6–11 weeks of age), predominant PRDC pathogens were PCMV (26.7%), PRRSV1 (25.5%), IAV-S (20.5%), and PCV-2 (11.4%). Other pathogens, such as PRRSV2, PRRSV1,2, and PRCV had a rather low prevalence (<3.0%). Prevalence of *A. pleuropneumoniae* rose from 24.7 to 32.1% during the post-weaning period. 

During the fattening period (12–25 weeks of age), prevalence of several PRDC pathogens showed a clear shift. PRRSV1 prevalence remained stable at 28.5%, although other PRRSV strain types also gained importance with PRRSV2 at 5.3% and PRRSV1,2 at 1.9% prevalence. PRCV and PCMV prevalence further decreased to 1.2% and 6.8%, respectively. Prevalence of IAV-S decreased to 11.4%, whereas PCV-2 increased to 27.1%. Prevalence of *A. pleuropneumoniae* further increased towards 47.9% during the fattening period.

Prevalence of different double and triple PRDC major pathogen interactions are given in Table 1. Most prevalent pathogen combinations among the different age categories are PRRSV–*M. hyopneumoniae*, PRRSV–IAV-S, PRRSV–PCV-2 and *M. hyopneumoniae*–IAV-S within the double infections and PRRSV–*M. hyopneumoniae*–IAV-S and PRRSV–*M. hyopneumoniae*–PCV-2 within the triple infections.

### 2.2. Seasonal Variation in Prevalence of M. hyopneumoniae and Other PRDC Pathogens at Piglet Level

Effect of season on prevalence of different PRDC pathogens or combinations of pathogens in different age categories are given in Table 2. 

At 3–5 weeks of age, no seasonal effect on prevalence of *M. hyopneumoniae* and other PRDC pathogens could be observed. At 6–11 weeks of age, several pathogens had a seasonal variation in occurrence. *Actinobacillus pleuropneumoniae* had the highest prevalence during summer (S3), whereas IAV-S was most prevalent during autumn (S4). For the viral agents PRRSV1 and PCMV, the highest prevalence occurred during winter (S1).

During the fattening period, both single PRDC pathogens and combined infections, including *M. hyopneumoniae*, showed seasonal variations. These combined infections included *M. hyopneumoniae*–PRRSV1 (7.2%), *M. hyopneumoniae*–PCV-2 (5.7%), *M. hyopneumoniae*–IAV-S (3.7%) and *M. hyopneumoniae*–PRRSV1–PCV-2 (3.3%). For most pathogens, highest prevalence could be observed in winter (S1), except for PCMV (autumn, S4), *A. pleuropneumoniae*, and the combined infection of PRRSV1–*A. pleuropneumoniae* (summer, S3). 

### 2.3. Impact of Climatological Parameters on Piglet Positivity for M. hyopneumoniae and Other PRDC Pathogens

The most relevant associations (expressed as odds ratio (OR) with the 95% confidence intervals between brackets) between prevalence of different pathogens during the 5-year study in Belgium and the Netherlands and local weather parameters are presented in Table 3.

Wind speed in the 10 weeks prior to sampling (WS_.10w_) and average wind speed in the 10 weeks prior to sampling (WS_avg.10w_) were only positively associated with all pathogen prevalence with OR ranging from 1.280 (PRRSV1) to 1.918 (PCV-2) for WS_.10w_ and OR ranging from 1.267 (PRRSV1) to 1.947 (*M. hyopneumoniae*–PCV-2) for WS_avg.10w_.

The difference between minimum and maximum outside temperature in the 10 weeks prior to sampling (T_diff_._10w_) was negatively associated with PRRSV1 (OR = 0.921), PCV-2 (OR = 0.834), *M. hyopneumoniae*–PCV-2 (OR = 0.818), and PRRSV–PCV-2 (OR = 0.799).

Duration of sunshine in the 10 weeks prior to sampling (S_dur.10w_) was positively associated with *A. pleuropneumoniae* (OR = 1.164) and negatively associated with PRRSV1 (OR = 0.911) and PRRSV–PCV-2 (OR = 0.817).

Minimal relative humidity in the 10 weeks prior to sampling (RH_min.10w_) was negatively associated with *A. pleuropneumoniae* (OR = 0.940), and positively associated with PRRSV1 (OR = 1.020), PCV-2 (OR = 1.030), and PRRSV–PCV-2 (OR = 1.048).

Wind direction in the 10 weeks prior to sampling (WD.10w) was negatively associated with PRRSV1 (OR = 0.981) and positively associated with *M. hyopneumoniae*–PCV-2 (OR = 1.026).

## 3. Discussion

Porcine respiratory disease complex remains one of the most important health concerns with a high economic impact for pig producers worldwide. The disease involves multiple viral and bacterial pathogens together with several non-infectious factors, such as ventilation, housing conditions, and management, leading to respiratory distress in pigs ranging from the peri-weaning period (3–5 weeks of age) over the post-weaning period (6–11 weeks of age) to the finishing stage (12–25 weeks of age). Interaction between both infectious (viral and bacterial agents) and non-infectious factors may all contribute to the development and severity of the respiratory disease [23]. The most commonly identified pathogens are PRRSV, IAV-S, PCV-2, and *M. hyopneumoniae*, besides other pathogens associated with PRDC, such as *Streptococcus suis*, *A. pleuropneumoniae*, *P. multocida*, DNT-positive *P. multocida*, *Glaeserella parasuis* (*G. parasuis*), *Mycoplasma hyorhinis* (*M. hyorhinis*), *Mycoplasma hyosynoviae*, PRCV, and PCMV [3,23,48].

Detection of the etiologic agents of PRDC has long been difficult, especially due to the wide variety of diagnostic approaches applied in practice. Diagnosis of *M. hyopneumoniae* could be performed using clinical signs, slaughterhouse checks of affected lungs [49,50], serological examination of relevant age groups [49,50], direct pathogen identification through bacteriological culture [44] or PCR techniques [51,52]. As for other respiratory pathogens involved in PRDC, more or less the same diagnostic approach has been applied, mainly due to lack of diagnostic tests able to simultaneously detect multiple respiratory pathogens in a single-reaction method [6]. Although these single pathogen detection techniques may be reliable and sensitive, they remain time-consuming, labor-intensive and, therefore, quite expensive. Moreover, for bacterial pathogens detection typically depends on culture-based methods that can take up to several days to obtain the final results.

Polymerase chain reactions and real-time PCR tests have been developed for several pathogens involved in PRDC and are characterized by their high sensitivity and ease of use. In combination with a reliable sampling technique, such as TBS, these detection methods based on PCR have been able to detect *M. hyopneumoniae* at an early age [45,46] and in an early stage of infection [53,54].

The results from the current study in 974 pig farms clearly demonstrate that pigs can be infected at an early stage with *M. hyopneumoniae*, which is in accordance with previous reports applying the same sampling technique [46]. However, besides *M. hyopneumoniae*, several other PRDC pathogens may be involved in the clinical picture of coughing piglets at 3–5 weeks of age, such as IAV-S, PRRSV1, PRCV, and PCMV, whereas the prevalence of PCV-2 is rather low at that stage. From a bacterial perspective, *A. pleuropneumoniae* is present in a quarter of the pigs sampled. These observations are in accordance with Sunaga et al. (2020) [6] who observed mixed infection with several bacterial (*A. pleuropneumoniae*, *Bordetella bronchiseptica*, *P. multocida*, *M. hyopneumoniae*, *M. hyorhinis*), and viral agents (PCV-2, PCMV, PRRSV US strain, PRCV) in some of the farms included in the study. Comparison of prevalence data is however not possible due to the low sample number (n = 6 farms, n = 30 samples) in this study [6] as compared to our study (n = 974 farms, n = 22,266 samples).

In contrast to previous studies [45,46,53,54], where sampling was focused on *M. hyopneumoniae* detection and prevalence only, the current study clearly demonstrates that *M. hyopneumoniae* is in many cases combined with other pathogens related to PRDC. The demonstrated presence of *M. hyopneumoniae* at an early stage (pre-weaning or post-weaning) might significantly impact the clinical course of other PRDC pathogens such as PRRSV [9,10], IAV-S [2,11,12], or PCV-2 [14,15].

In the post-weaned piglets (6-11 weeks of age), some clear differences in PRDC pathogen prevalence could be observed. *M. hyopneumoniae* and both PRRSV1 and PRRSV2 doubled in prevalence as compared to peri-weaned piglets (3–5 weeks of age), together with PCMV and *A. pleuropneumoniae* that showed a more moderate increase. In contrast, PRCV rapidly decreased during the post-weaning period. The prevalence of PCV-2 increased over four times during the post-weaning phase, indicating that PCVAD became more prominently involved in respiratory problems during this phase. Besides single infections with one of these PRDC pathogens, several combined double and triple infections (Table 1) could also be detected involving the major pathogens PRRSV, *M. hyopneumoniae*, PCV-2, and IAV-S and *A. pleuropneumoniae* as the most prevalent bacterial agent. This implies that piglets in the post-weaning phase are exposed to mixed infections of several viral pathogens known as immunosuppressive, leading to a compromised immune response towards vaccination or other concurrent bacterial infections such as post-weaning diarrhea due to enterotoxigenic *Escherichia coli*.

During the fattening phase, the most prominent PRDC pathogens in pigs with clinical signs of respiratory disease were *M. hyopneumoniae*, PRRSV1, PRRSV2, and PCV-2, together with *A. pleuropneumoniae* and to a lesser extent IAV-S. The high prevalence of *M. hyopneumoniae* is of particular interest and may be partially explained by the long duration of pathogen persistence in the respiratory tract of the infected pigs. Previous studies have detected *M. hyopneumoniae* up to 254 days post-infection in deep laryngeal swabs using a PCR technique [55]. Recent research into specific risk factors influencing *M. hyopneumoniae* infection status revealed that both age and type of production system had an impact on infection rate and pathogen prevalence [56]. Infection rates were higher in older animals and the prevalence was higher in the one- and two-site systems than in the three-site systems. Dynamics of infection by RT-PCR showed that *M. hyopneumoniae* infection on one-site farms occurs earlier, while on two- and three-site farms occurs later but spreads faster, suggesting that contact between animals of different age favors the transmission. However, in the present study, data on the type of production system were not recorded. Nevertheless, the impact of age was similar in our study, since the prevalence of *M. hyopneumoniae* is substantially higher in the fattening pigs as compared to peri-weaned or post-weaned piglets with clinical signs of respiratory disease [56]. Therefore, piglets infected with *M. hyopneumoniae* during the post-weaning period or the early fattening period can excrete the pathogen for the entire fattening period, thus, increasing the probability of detection when pigs are sampled during an episode of respiratory problems. This is in contrast with other pathogens such as IAV-S that have a rather short period (max. 6–8 days) of detection at the level of the respiratory tract [57,58]. The results obtained concerning PCV-2 dynamics in the current study are in accordance with a recent study on serological and viral dynamics of PCV-2 carried out in PCV-2 infected pig herds in Taiwan [59]. As reported previously, we also observed an increased prevalence during the grow (11.2%) and finish phase (27.1%) [59].

In the peri-weaned piglets, no effect of season on prevalence of PRDC pathogens could be observed. Presence of PRDC pathogens at that age is mainly determined by the infection status of their dam and outside conditions have very little impact on infection status at that age. In contrast, in post-weaned piglets, single PRDC pathogens are affected in their seasonal prevalence, with *A. pleuropneumoniae* at the highest level in summer (S3), IAV-S in autumn (S4) and both PRRSV1 and PCMV in winter (S1). In practice, it is well-known that respiratory problems related with *A. pleuropneumoniae* frequently occur at the change of seasons, especially with warmer days and colder nights, as might happen in our region at start of spring (April/May; S2) and the end of summer (August/September; S3), which explains the higher prevalence in these two seasons in our study. On the contrary, IAV-S occurs later in the year, when weather conditions turn cold and wet, which is the case in autumn (S4). In the colder winter months, PRRSV1 and PCMV seem to circulate more pronouncedly. The same pattern continues in the older pigs during the fattening period (12–25 weeks of age), where most PRDC pathogens and several combined respiratory infections are highly prevalent during winter (S1). Only exceptions from this pattern are PCMV (S4) and infections involving *A. pleuropneumoniae*, which are again more prominent during late summer (S3), as previously discussed.

Several climatological parameters were significantly associated with PRDC pathogen prevalence. The most prominent parameters are relative humidity (RH_min.10w_), temperature difference between minimum–maximum temperature (T_diff.10w_), wind speed (WS_.10w_), average wind speed (WS_avg.10w_), wind direction (WD._10w_) and duration of sunshine (S_dur.10w_). Apparently, the weather conditions in the 10 weeks prior to sampling have the largest impact on pathogen prevalence. Another interesting observation is that from all PRDC pathogens detected in prevalences higher than 5%, prevalence of *M. hyopneumoniae* and PRRSV is most frequently impacted by weather conditions. In a Spanish study [60], the influence of climatological parameters on *M. hyopneumoniae* dynamics was also explored. This study revealed the higher the precipitation rate, the higher the probability of being *M. hyopneumoniae* nPCR-positive on nasal swabs, whereas the lower the temperature, the higher the probability of being *M. hyopneumoniae* seropositive. From a seasonal perspective, animals born in autumn and reaching slaughter in spring had the highest probability of being infected by *M. hyopneumoniae* and the highest probability of being *M. hyopneumoniae* seropositive [60]. Moreover, bio-aerosols, containing *M. hyopneumoniae* and PRRSV, were capable of spreading pathogens between herds via airborne route. Conditions common to both pathogens include cool temperature and specific wind direction, and more specifically low sunlight levels, low wind velocity in combination with rising humidity and air pressure [61].

The present study clearly shows that *M. hyopneumoniae* and different viral and bacterial pathogens responsible for PRDC may be present during the peri-weaning, post-weaning, and fattening period. Following analysis of seasonal variation, it can be concluded that depending on the pathogen, a clear variation in seasonal impact on the prevalence of PRDC pathogens is present. Moreover, several climatological parameters may influence the prevalence of the detected PRDC pathogens.

## 4. Materials and Methods

### 4.1. Selection of Study Herds

The study was conducted from September 2011 to September 2016 in Belgium and the Netherlands. Closed pig herds were selected through regular contacts with local veterinary practices. Inclusion criteria were as following: at least 200 sows, at least two age groups (3–5 weeks of age, 6–11 weeks of age, and 12–25 weeks of age) available for sampling, presence of clinical signs of respiratory disease (coughing), and no use of antimicrobials active against *M. hyopneumoniae* in piglets less than 3 weeks of age and during the last 2 weeks prior to sampling in the post-weaning or fattening phase. Piglets or fattening pigs eligible for sampling were marked up by the swine farmer or herd veterinarian prior to sampling. In total, 974 closed pig herds were included in the study, equally distributed over different seasons of the year (Table 4). In total, 22,266 pigs with clinical signs of respiratory disease were sampled. On average, 22.8 pigs were sampled per herd with a minimum of 10 pigs and a maximum of 30 pigs, distributed over the different age categories that suffered from clinical signs of respiratory disease. Therefore, samples were collected during the peri-weaning (3–5 weeks of age), post-weaning (6–11 weeks of age), and fattening (12–25 weeks of age) period. The latter group was only sampled in case of presence of clinical signs of respiratory disease in that age group. Within each herd, we sampled piglets affected with clinical signs of respiratory disease from as many different compartments and pens in the nursery as possible. Sampling was always performed by the same veterinarian trained on TBS sampling.

### 4.2. Tracheobronchial Swab (TBS) Sampling Procedure

TBS sampling was performed as previously described [45,46]. Briefly, TBS were obtained through thorough fixation of the piglets with a nose snare, followed by use of a mouth opener. The TBS (aspiration tube, 50 cm, 12CH; Medinorm GmbH, Spiesen-Elversberg, Germany) was subsequently inserted through the mouth, through the glottis down to the tracheobronchial split. Mucus was collected through gentle movement of the swab at the level of the tracheobronchial split and the swab was subsequently retrieved. The tip of the swab was collected in a sterile tube (MLS, Menen, Belgium) with 1 mL of sterile saline solution (Saline Solution 0.9%; Eurovet, Heusden-Zolder, Belgium) and kept cool at 3 °C until analysis within 48 h after sampling.

### 4.3. Analysis of TBS Swabs

The material collected by the TBS was processed according to Strait et al. (2008) [62]. A multiplex PCR (mPCR) analysis was performed according to the standard operating procedure of the laboratory (IVD GmbH, Hannover, Germany). The mPCR included analysis of *M. hyopneumoniae*, PRRSV (including differentiation in European (PRRSV1) and North-American (PRRSV2) or combined European and North-American strain type (PRRSV1,2)), PCV-2, IAV-S, PCMV, and PRCV. A second PCR for bacterial species (*A. pleuropneumoniae*) was run on the same samples. The test was an App apxIV-PCR (IVD GmbH, Germany; in-house test; Strutzberg-Minder, 2009). It was a specific 414 bp-target, which had been tested for the detection of App serotype 1 to 19. Specificity was tested against the following species: *Glaesserella parasuis* serotypes 1–15, *Actinobacillus porcitonsillarum*, *Actinobacillus suis*, *Actinobacillus minor*, *Actinobacillus indolicus*, *Actinobacillus porcinum*, *Actinobacillus equuli*, *Actinobacillus lignieresii*, *Mannheimia haemolytica*, *Pasteurella multocida*, and *Streptococcus suis*. Analytical sensitivity was 1000 GE/mL. PCR results were reported as negative or positive for the presence of the different PRDC pathogens.

### 4.4. Data Categorization for Seasonality

In order to assess the association among season, as defined by calendar months, and infection dynamics of *M. hyopneumoniae* and other PRDC pathogens sampled, herds were categorized for seasonality based on date of sampling. Seasonality was implemented as the following: S1—winter (21/12–20/03; n = 227 herds), S2—spring (21/03–20/06; n = 247 herds), S3—summer (21/06–20/09; n = 213 herds), and S4—autumn (21/09–20/12; n = 287 herds) (Table 4).

### 4.5. Climatologic Data

Climatologic data collected are presented in Table 5. These data were collected from July 2011 to September 2016. Climatologic data were provided by the local meteorological institute (KNMI, Koninklijk Nederlands Meteorologisch Instituut; http://www.knmi.nl/klimatologie (accessed on 16 October 2016)).

Besides the climatologic data observed on the day of sampling, a rolling average of the data over a 1-, 2-, 4-, and 10-week period were calculated for all sampling days throughout the 5-year study period.

### 4.6. Statistical Analysis

The effect of season on the prevalence of PRDC pathogens was investigated with logistic regression mixed models with a random effect for herd. The overall effect of season was tested with the likelihood ratio test, and all pairwise comparisons between seasons were performed on the log odds ration scale with Wald tests [63] and with Bonferroni adjustment for controlling the familywise error rate (FWER) at 5%. These analyses were repeated for all pathogens and within the three age categories (3–5, 6–11, and 12–25 weeks of age).

The effects of the climate parameters on the prevalence were investigated by fitting logistic regression mixed models with herd as random effect. The models were fitted for each combination of pathogen and climate parameter. The significance of the effects was tested by means of Wald tests. Given the large number of tests, false positives were controlled with the method of Storey (2003) [64] for controlling the false discovery rate (FDR) at 5%.

All statistical analyses were performed in the statistical software R [65] (R Core Team, 2021) and the R packages lme4, emmeans, and qvalue. All hypothesis tests were performed at the 5% level of significance, and multiple testing procedures were performed at the 5% FWER or the 5% FDR level.

## 5. Conclusions

In conclusion, the present study showed that many respiratory pathogens are present during the peri-weaning, post-weaning, and fattening period, which may complicate the clinical picture of respiratory disease. Moreover, interactions between these PRDC pathogens, season, and local weather conditions could be demonstrated over the five-year study period.

## Figures and Tables

**Table 1:** Prevalence (expressed as % positive samples ± SEM) of different double and triple PRDC major pathogen interactions in different age categories, namely peri-weaned piglets (3–5 weeks of age), post-weaned piglets (6–11 weeks of age), and fattening pigs (12–25 weeks of age). In total, 22,266 pigs were sampled. PRRSV1, European strain of porcine reproductive and respiratory syndrome virus; PRRSV2, North-American strain of PRRSV; PRRSV1,2, combined European and North-American strain of PRRSV; *Mhyo*, *Mycoplasma hyopneumoniae*; IAV-S, influenza A virus in swine; PCV-2, porcine circovirus type 2; *App*, *Actinobacillus pleuropneumoniae*.

Pathogen Combination	Age Category		
	3–5 w	6–11 w	12–25 w
*Double infections*			
*PRRSV–Mhyo*	1.9 ± 0.5% ^a^	6.1 ± 0.8% ^b^	21.0 ± 1.3% ^c^
*PRRSV1–Mhyo*	1.8 ± 0.5% ^a^	5.2 ± 0.7% ^b^	15.9 ± 1.1% ^c^
*PRRSV2–Mhyo*	0.1 ± 0.1% ^a^	0.7 ± 0.3% ^a^	3.7 ± 0.6% ^a^
*PRRSV1,2–Mhyo*	0.0 ± 0.1% ^a^	0.2 ± 0.2% ^a^	1.3 ± 0.4% ^a^
*PRRSV–IAV-S*	3.3 ± 0.6% ^a^	5.1 ± 0.7% ^b^	2.8 ± 0.5% ^ab^
*PRRSV1–IAV-S*	3.1 ± 0.6% ^a^	4.5 ± 0.7% ^a^	2.4 ± 0.5% ^a^
*PRRSV2–IAV-S*	0.2 ± 0.2% ^a^	0.5 ± 0.3% ^a^	0.4 ± 0.2% ^a^
*PRRSV1,2–IAV-S*	0.0 ± 0.1% ^a^	0.2 ± 0.2% ^a^	0.0 ± 0.0% ^a^
*PRRSV–PCV-2*	1.0 ± 0.3% ^a^	5.4 ± 0.8% ^a^	9.5 ± 0.9% ^a^
*PRRSV–PCV-2*	0.9 ± 0.3% ^a^	4.4 ± 0.7% ^a^	7.7 ± 0.8% ^a^
*PRRSV2–PCV-2*	0.1 ± 0.1% ^a^	0.6 ± 0.3% ^a^	1.2 ± 0.3% ^a^
*PRRSV1,2–PCV-2*	0.0 ± 0.0% ^a^	0.3 ± 0.2% ^a^	0.7 ± 0.3% ^a^
*PRRSV–App*	1.0 ± 0.9% ^a^	2.7 ± 1.7% ^a^	4.8 ± 1.7% ^a^
*PRRSV1–App*	0.9 ± 0.9% ^a^	2.3 ± 1.3% ^b^	3.7 ± 1.5% ^c^
*PRRSV2–App*	0.0 ± 0.2% ^a^	0.3 ± 0.3% ^a^	0.6 ± 0.6% ^a^
*PRRSV1,–App*	0.0 ± 0.2% ^a^	0.1 ± 0.2% ^a^	0.5 ± 0.5% ^a^
*Mhyo–IAV-S*	2.7 ± 0.6% ^a^	2.5 ± 0.5% ^a^	5.4 ± 0.7% ^b^
*Mhyo–PCV-2*	0.8 ± 0.3% ^a^	3.3 ± 0.6% ^a^	17.1 ± 1.2% ^a^
*Mhyo–App*	0.7 ± 0.7% ^a^	1.9 ± 0.7% ^b^	7.6 ± 1.2% ^c^
*Triple infections*			
*PRRSV–Mhyo–IAV-S*	0.4 ± 0.2% ^a^	0.8 ± 0.3% ^a^	1.6 ± 0.4% ^a^
*PRRSV1–Mhyo–IAV-S*	0.3 ± 0.2% ^a^	0.8 ± 0.3% ^ac^	1.5 ± 0.4% ^bc^
*PRRSV2–Mhyo–IAV-S*	0.0 ± 0.1% ^a^	0.0 ± 0.1% ^a^	0.1 ± 0.1% ^a^
*PRRSV1,2–Mhy –IAV-S*	0.0 ± 0.1% ^a^	0.0 ± 0.0% ^a^	0.0 ± 0.0% ^a^
*PRRSV–Mhyo–PCV-2*	0.3 ± 0.2% ^a^	1.7 ± 0.4% ^a^	6.9 ± 0.8% ^a^
*PRRSV –Mhyo–PCV-2*	0.3 ± 0.2% ^a^	1.3 ± 0.4% ^b^	5.4 ± 0.7% ^c^
*PRRSV2–Mhy –PCV-2*	0.0 ± 0.1% ^a^	0.2 ± 0.2% ^a^	0.9 ± 0.3% ^a^
*PRRSV1,2–Mhy–PCV-2*	0.0 ± 0.0% ^a^	0.1 ± 0.1% ^a^	0.7 ± 0.3% ^a^
*PRRSV–IAV-S–PCV-2*	0.3 ± 0.2% ^a^	0.7 ± 0.3% ^a^	0.5 ± 0.2% ^a^
*PRRSV1–IAV-S–PCV-2*	0.2 ± 0.2% ^a^	0.7± 0.3% ^a^	0.5 ± 0.2% ^a^
*PRRSV2–IAV-S–PCV-2*	0.0 ± 0.1% ^a^	0.0 ± 0.1% ^a^	0.0 ± 0.0% ^a^
*PRRSV1,2–IAV-S–PCV-2*	0.0 ± 0.0% ^a^	0.0 ± 0.1% ^a^	0.0 ± 0.0% ^a^

Significant differences (*p* < 0.05) in pathogen prevalence are indicated by different letters (a–c) in superscript.

**Table 2:** Effect of season on prevalence of different PRDC pathogens or PRDC pathogen combinations in different age categories. Pathogen prevalences are given in %. Significant differences (*p* < 0.05) are indicated with different letters in superscript. PRRSV1, European strain of porcine reproductive and respiratory syndrome virus; PRCV, porcine respiratory coronavirus; PCMV, porcine cytomegalovirus; *M. hyopneumoniae*, *Mycoplasma hyopneumoniae*; IAV-S, influenza A virus in swine; PCV-2, porcine circovirus type 2; *A. pleuropneumoniae*, *Actinobacillus pleuropneumoniae*. Seasons with the highest pathogen prevalence are in red.

Age Category	Pathogen	Season			
		S1	S2	S3	S4
6–11 w	PRRSV1	14.2 ^b^	10.3 ^ab^	8.2 ^a^	13.0 ^ab^
	IAV-S	23.5 ^a^	32.8 ^b^	21.3 ^a^	33.9 ^b^
	PCMV	21.4 ^b^	18.4 ^ab^	14.8 ^a^	18.3 ^ab^
	*A. pleuropneumoniae*	20.2 ^ab^	31.0 ^b^	32.6 ^b^	15.0 ^a^
12–25 w	PRCV	1.8 ^ab^	2.2 ^b^	0.7 ^a^	1.5 ^ab^
	PRRSV1	35.5 ^b^	24.3 ^a^	22.8 ^a^	21.5 ^a^
	PCMV	25.6 ^ab^	26.9 ^bc^	21.9 ^a^	31.1 ^c^
	PCV-2	16.3 ^c^	12.8 ^bc^	9.2 ^ab^	8.2 ^a^
	*A. pleuropneumoniae*	30.5 ^b^	37.1 ^bc^	45.2 ^c^	18.0 ^a^
	PRRSV1/*M. hyopneumoniae*	7.2 ^b^	4.8 ^ab^	5.5 ^ab^	3.7 ^a^
	PRRSV1/PCV-2	8.1 ^b^	3.9 ^a^	3.9 ^a^	3.6 ^a^
	PRRSV1/*A. pleuropneumoniae*	2.2 ^ab^	2.3 ^ab^	4.0 ^b^	1.2 ^a^
	*M. hyopneumoniae*/IAV-S	3.7 ^b^	2.0 ^ab^	1.5 ^a^	2.8 ^ab^
	*M. hyopneumoniae*/PCV-2	5.7 ^b^	3.5 ^ab^	2.2 ^a^	2.3 ^a^
	PRRSV1/*M. hyopneumoniae*/PCV-2	3.3 ^b^	0.7 ^a^	1.0 ^a^	0.6 ^a^
	PRRSV1/IAV-S/PCV-2	1.4 ^b^	0.6 ^ab^	0.6 ^ab^	0.3 ^a^

Significant differences (*p* < 0.05) in pathogen prevalence are indicated by different letters (a–c) in superscript.

**Table 3:** Associations (OR—odds ratio) between overall pathogen prevalence during the five-year study in Belgium and the Netherlands and specific weather parameter registered at the weather stations located nearby the sampled farm included in the study. Only significant associations (Q < 0.001) for pathogens with an overall prevalence in all three age categories >5% are given. PRRSV, porcine reproductive and respiratory syndrome virus; PRRSV1, European strain of PRRSV; *Mhyo*, *Mycoplasma hyopneumoniae*; PCV-2, porcine circovirus type 2; *App*, *Actinobacillus pleuropneumoniae*. WS—wind speed; WS_avg_—average measured wind speed; T_diff_—difference between maximal and minimal temperature measured over the day; S_dur_—duration of sunshine; RH_min_—minimal relative humidity and WD—wind direction. Co-notations associated with the weather parameter indicated the period: 10 w—10-week rolling average. Positive associations have an OR > 1, negative associations have an OR < 1.

Pathogen	WS.10w	WS_avg_.10w	T_diff_.10w	S_dur_.10w	RH_min_.10w	WD.10w
*App*				1.164	0.940	
				(1.063; 1.274)	(0.911; 0.970)	
PRRSV1	1.280	1.267	0.921	0.911	1.020	0.981
	(1.126; 1.456)	(1.116; 1.440)	(0.880; 0.965)	(0.861; 0.964)	(1.009; 1.032)	(0.970; 0.992)
PCV-2	1.836	1.832	0.834		1.030	
	(1.516; 2.225)	(1.517; 2.214)	(0.777; 0.896)		(1.012; 1.049)	
*M. hyopneumoniae*—PCV2	1.918	1.947	0.818			1.026
	(1.460; 2.520)	(1.484; 2.553)	(0.736; 0.911)			(1.012; 1.040)
*M. hyopneumoniae*—PRRSV	1.404					
	(1.150; 1.714)					
PRRSV—PCV-2	1.753	1.738	0.799	0.817	1.048	
	(1.327; 2.316)	(1.317; 2.293)	(0.718; 0.889)	(0.721; 0.925)	(1.021; 1.075)	

**Table 4:** Number of swine farms in Belgium and the Netherlands sampled per year and per season during the entire study period from S4 2011 until S3 2016, including total number of sampled farms per year and per season. S1, winter; S2, spring; S3, summer; and S4, autumn.

Season	Year						Total/Season
	2011	2012	2013	2014	2015	2016	
S1		51	53	44	34	45	227
S2		40	40	53	45	69	247
S3		31	41	47	32	62	213
S4	47	50	70	69	51		287
Total/Year	47	172	204	213	162	176	974

**Table 5:** List of climatological observation parameters with their abbreviations used and their specific units.

Parameter	Abbreviation	Units
WD	Wind direction	°
WS	Wind speed	0.1 m/s
WS_avg_	Average wind speed over 12 h	0.1 m/s
T	Average temperature over 12 h	0.1 °C
T_min_	Minimum temperature	0.1 °C
T_max_	Maximum temperature	0.1 °C
T_diff_	Difference maximal–minimal temperature measured over the day	0.1 °C
S_dur_	Duration of sunshine	0.1 h
P_dur_	Duration of precipitation	0.1 h
P	Total precipitation in 12 h	0.1 mm
AP	Average air pressure at sea level over 12 h	0.1 Pa
RH	Relative humidity	%
RH_max_	Maximal relative humidity	%
RH_min_	Minimal relative humidity	%

## Data Availability

The data presented in this study are available upon request from the corresponding author.
